# Book Reviews

**Published:** 1820-02

**Authors:** 


### CRITICAL ANALYSIS

OF

### RECENT PUBLICATIONS, IN THE DIFFERENT BRANCHES OF MEDICINE AND SURGERY;

### SELECT MEMOIRS, AND HISTORIES OF CASES;

### In the Literature of Foreign Nations.

Πατρίδες ἄρα
Ανδράσιν, ὧ πατρὰς ἄνδρες, ἀγαλλόμεθα.

### *Exposition of the Doctrine of* M. Broussais.

[*Continued from page* 68.]

BEFORE we proceed to the particular application of the facts detailed in the preceding article, it may be proper to repeat some remarks made in it, relating to the different consequences resulting from the same causes in different individuals; as they tend to explain several difficulties in the history of some diseases that have led pathologists to suppose the existence of some latent vice to which they owe their immediate origin. It is the superiority of the development of the nervous and sanguiferous systems, which, according to M. Broussais, disposes certain persons to the neuroses, to acute inflammations, and to hæmorrhages; whilst an analogous constitutional disposition, but in which the lymphatic system enjoys a remarkable predominance of action, is the organic condition which renders other persons so readily liable to morbid enlargement of the lymphatic glands, and the degeneration of structure which we have already particularly described. There are many persons who are remarkable for the little energy with which the circulation is effected in them: these are all disposed to irritations of the lymphatic vessels and ganglions, and to those degenerescences of structure which have been termed organic lesions; whilst the same causes to which they owe their origin, will, in persons whose circulation is carried on with greater vigour, give rise to violent phlegmasiæ. Those degenerescences have been shown to bear a near analogy to each other: nearly the whole of them have been observed in almost every part of the animal economy; but they, or the symptoms resulting from them, have received different names from nosologists, according as they may have their seat in particular organs. There is this analogy especially between tubercular phthisis, scrofula, and the affection causing tabes mesenterica.

The causes of enlargement of the lymphatic glands all act, either by irritating the glands themselves, or by exciting the surfaces on which the extremities of the absorbent vessels communicating with them terminate. The former is the less frequent, and cannot indeed exert its influence on the glands contained in the great cavities of the body: but the latter is very frequent, and is, as we shall presently show, according to M. Broussais, the general cause of phthisis. Inflammation of any organ produces enlargement of the glands whose vessels originate from the inflamed part; and, if the excitement thus effected becomes chronic, the glands remain enlarged, they pass to the tuberculous state, and, at length, the same degenerescence takes place, from the laws of association so remarkable between the tissues of the same structure and properties, in the glands of other parts of the body, so as to produce a diathesis analogous to the scrofulous. It has been denied that lymphatic glands, that have become swelled from sympathy, ever become tuberculous; but M. Broussais asserts, that he has witnessed it in the exterior parts of the body in the most indubitable manner: and thence it is that inflammation of the mucous membrane of the lungs is a very frequent, and indeed almost the exclusive, cause of tubercular phthisis. The same origin applies to tabes mesenterica. The signs of irritation of the stomach and bowels, which are evident throughout its whole duration; the causes which determine it; the results of the examination of dead bodies; the good effects of abstinence from food, of cooling drinks, and of revulsives, conjoin to prove, says M. Broussais, that tabes mesenterica is nothing else than irritation of the mucous membrane of the gastric canal, especially of the small intestines. (The enlarged glands, it is said in the Lectures, correspond especially to the parts of the intestine in which the inflammation had been most severe.) This irritation, which is similar to that in gastro enteritis in general, produces with more facility that enlargement of the mesenteric glands in children, because those organs are in them much developed, and enjoy a greater relative degree of vital energy than at any other period of life.

We return to the consideration of phthisis in a more particular manner. M. Broussais has shown that this affection is the almost inevitable consequence of prolonged irritation of the mucous membrane of the lungs in lymphatic subjects; and he adduces the most powerful evidence to show that this is its almost exclusive origin. During the twelve years that he accompanied the French army through the principal countries on the continent of Europe, he had frequent occasion to witness the confirmation of the above statement, and, at the same time, of determining the influence of a cold and moist climate in its production.

When I arrived at the army in Holland, he says, I found phthisis so frequent there, that, whenever a catarrh, accidentally arising from cold, was prolonged in a fair, slender, and lymphatic subject, it was necessary, in a manner, to despair of his recovery. On opening those patients, the lungs were found studded with tubercles and granulations. M. Broussais then suspected that, if those patients had lived in a warmer climate, and not contracted the inflammation which marked the origin of the disease, they would not have fallen victims to phthisis; and that, if men of the same constitution, who were not yet affected with any disease, continued to be exposed to cold, they might, in contracting pulmonary inflammation, have the fate of their comrades; whilst, if they passed into a warm climate, they would escape that fatal disease. This supposition became realized: the army was transported into Italy; phthisis then became scarce, exactly in proportion to catarrh and pneumonia. Here, and afterwards in the south of Spain,

M. Broussais opened a great number of the bodies of subjects of the constitution termed phthisical, who had died of other affections; and he never found tubercles in the lungs, except in a very few instances, where that organ had been irritated for a long time before death: that is to say, when the patients had suffered as they would have done in a colder country. These statements furnish powerful arguments against the opinions of those who suppose the tubercles to be the exciting cause of the inflammation; especially with the additional one, that, after twelve years' experience, M. Broussais says he saw but very few men fall into phthisis, who had not, even under his own observation, been affected with prolonged catarrhs, or peripneumonia.

The various appearances offered by the tuberculous and other degenerescences described in the preceding article, are commonly witnessed in the lungs, and not unfrequently at the same time;—a circumstance arising from their complex structure, into which nearly all the elementary tissues of the body enter. It is hardly necessary to remark, that the same irritative cause will produce different effects in each of those tissues, from the difference in their structure, and from its longer or shorter duration. Thus, we sometimes find in the lungs, indurations, some of a white, others of a yellowish, colour, in masses more or less considerable in size, and presenting appearances more or less analogous with what is termed schirrous and tuberculous matter; in the midst of which, calculous and osseous concretions have been observed. In other subjects, there is a confused mass of black, grey, and yellowish, spots, forming so many globules, some of them hard, and others friable: the fluid they have contained has shown the same varieties of character, being sometimes purulent, sometimes sanious, at others reddish and cream-like, &c. Ulcers, and bands of cellular tissue, traversing the lungs in various directions, are other varieties of organic lesion here observed. M. Broussais has not been able to determine precisely the causes of those varieties, which have furnished nosologists with the bases for so many distinct species of the disease. Some of the more strongly-marked characters may, however, be probably referred to the following laws: 1°. Active inflammation, affecting chiefly the sanguiferous vessels, will make the carnification of the lungs prevail; but this may not be the only alteration of structure, if the organs have been long in a state of irritation. 2°. A general disposition to irritation of the lymphatics, proved by other affections entirely of this nature, will lead to a predominant tuberculous disorganization. 3°. There will also be an affection of the same organs, happening as a consequence of irritation ofthe lymphatics of the cellular texture, which constitutes schirrus, when those irritations have existed for a long time in some external organ.

The scrofulous diathesis is, according to M. Broussais, merely the lymphatic constitution carried to the highest degree, which may be transmitted from parents to their children, just as the sanguineous and nervous temperaments may be so transferred; but scrofula cannot thence be said to be a latent disease in persons of that constitution, any more than pleurisy, or hepatitis, or other acute inflammatory affection, to which those of the sanguineous temperament are so especially liable, can be said to exist in these as constitutional diseases. This is reducing what is called the *scrofulous habit* to its true character.

In the treatment of *scrofula*, the first indication which presents itself, when the patient only shows a disposition to that malady, is to combat this disposition by hygienic measures. We cannot then act immediately on the lymphatic system to diminish its action, we must, therefore, endeavour to establish a more near equilibrium, by giving to the sanguineous system an energy which may remove the relative preponderance of the lymphatic vessels. This may be attempted by moderate exercises, especially in the open air, generous diet, wine, &c.: but, in giving this impulse to the system, it will be necessary to avert carefully all causes of more especially local irritation; the susceptibility of the stomach to excitation must be well attended to, and cold and humidity avoided. When the glands on the exterior parts of the body are tumefied, the same means may be persisted in; because this irritation very frequently arises from external causes, especially change of temperature, or from some part of the absorbent system being irritated in the manner, and with the results, described on a former occasion, (page 64). But this is not the case with the glands of the lungs and mesentery: here, according to M. Broussais, it is irritation of the mucous surfaces on which the vessels communicating with them open, which has been the origin of the affection of the glands. Here it is the inflammation of the membranous surfaces that requires our principal care, in the first instance; but, in proportion as that inflammation subsides, the treatment of the enlargement of the glands that may be suspected to remain, should approximate to the mode already indicated for scrofula, when affecting the lymphatics of the exterior parts of the body. But this new indication, which appears only second in order in inflammatory phthisis, the causes of which are most evident, becomes primitive and fundamental in those where the phlogosis is developed only consecutively to the lymphatic disorder. The basis of the treatment, according to the former indication, should be placed on the therapeutics for catarrh, peripneumonia, and pleurisy; and, if the inflammation assumes a chronic form, it will be necessary to avoid the occasional causes, as cold and humid air, or any other irritating cause depending on the habits of life of the patient. The measures, speaking generally, are blood-letting, in the first instance, with restricted diet, and other analogous sedative measures; then revulsives; and, at length, a recourse to means proper to balance the vitality between the sanguiferous and lymphatic systems. These measures will require modification, according to the particular forms of disease under which the irritation of the lymphatics has its origin: 1°. When *pneumonia* is the cause, the antiphlogistic measures should be directly debilitant. Free blood-letting will be necessary. The revulsives appropriate to it, are those which evacuate distant vessels without irritating them. The regimen must be very restricted.

2°. The *catarrhal phthisis* requires a combination of antiphlogistics, sedatives, and revulsives, restricted diet in the first instance, then nourishing without being stimulant. In the first instance, the action of the sanguiferous system should be moderated by general or local bleeding, according to particular circumstances; cooling drinks, and abstinence from food ; cutaneous transpiration should be favoured by the pediluvium, during the violence of the erethism ; and, when this diminishes, by vesicatories and rubefacients. As soon as white and thick expectoration announces a resolution of the inflammation, tonics may be combined with emollients, the patient may be permitted to take food, and gradually return to his ordinary mode of life. When patients have previously been in a state of great exhaustion, the resolutive expectoration either does not take place, or it is too far prolonged. Thus, the chronic state appears under two forms : 1°, with dry cough, or but a little occasional spitting of transparent mucus; 2°, with abundant expectoration of thick and opaque matter. The patient's diet should here be nourishing, and wine and other tonics directed; only observing that he eats but little towards the evening, lest some febrile exacerbation should be then induced. A weak decoction of cinchona, combined with mucilages, has appeared to M. Broussais to be the most proper medicine.

3°. *Pleuritic phthisis* does not, unless the patient be plethoric, require the opening of the large veins, like the pneumonic : cupping, and the application of leeches, are more appropriate, followed by the tepid bath, emollient applications over the chest, to encourage the oozing of blood after the leeches have been removed; and slightly diaphoretic drinks. Vesicatories, until there are evident signs of alleviation, M. Broussais considers, are not advisable; principally because they often lead the practitioner to suppose that the inflammation is removed, when it exists with its original violence. In many cases, he found the pain in the chest disappear on the production of a blister externally, and yet the patients have died after a few days. Dissection showed that the inflammation had persisted with its original intensity, although the pain, considered so constant a symptom of it, had been totally removed. The diet should be aqueous, or vegetable ; and the treatment indicated be continued until the most complete re-establishment of the functions: there is nothing else can attest that the inflammation of the membrane is entirely removed. From a want of due attention to this, many patients have fallen into consumption; the slight uneasiness about the chest being considered only as an indolent consequence of the previous inflammation, when it has really arisen from a less intense degree of that disorder. The utmost quiet of body, the avoiding of cold air, the use of tepid baths, and of flannel worn next the skin, with antiphlogistics adapted to the degree of the complaint, must be directed. It is this class of medicines which generally are the most efficacious against the cough that may remain ; but, when this appears to be kept up by an irritation of the mucous membrane of the larynx and bronchiæ, and is not calmed by the measures indicated, the use of opium remains: " it is always the best resource against those irritations of the chest which refuse to give way to antiphlogistics, and which are exasperated by irritatant revulsives."

4°. The *phthisis dependant on the profession and habits of life*, requires the avoidance of the exciting causes, and the application of the foregoing measures, adapted to the particular circumstances of the individual case.

5°. *Phthisis consequent on continued fevers*, or, rather, the irritations of the lungs which succeed to those diseases, and which, by their obstinacy, lead to a fear of the formation of tubercles, requires some distinction in its treatment: 1°, if the inflammation is vehement, and the patient but little exhausted, the most rigorous antiphlogistic regimen, especially almost total abstinence from food, although it may be urgently called for by the appetite; besides this, revulsive measures proportionate to the excitability of the subject: 2°, if the fever has produced much debility, if the inflammation be languid, obscure, or if an abundant expectoration exists, sudorifics and revulsive irritants are well indicated; observing, however, that any febrile action that may remain be not augmented by them. The effects of those means should be confined to the secretory, or excretory, capillary vessels; the actions of which we wish to augment, in order to destroy the disposition which directs the fluids towards the lungs. In some cases of this kind, it has, however, been more advantageous to introduce into the stomach some astringent tonic, the effects of which are opposite to those of the foregoing agents,—such as cinchona. Morton relates instances of surprising cures effected by this bark, in obstinate coughs with hectic fever, and very abundant expectoration indicating suppuration. This success has also been obtained by many other practitioners, and by M. Broussais. The cases in which this remedy is appropriate, may be thus generalized: (*a*) those consequent on diseases which have produced considerable debility in a short time,—as typhous fevers, excessive hæmorrhages, &c.; (*b*) when the patient is of a lax, feeble, lymphatic, temperament, and the stomach rather indolent; (*c*) in persons living in cold and humid countries, and in large populous cities, rather than in warm latitudes, exposed situations, and villages.

6°. *Scorbutic phthisis*, or chronic irritation of the lungs modified by the scorbutic diathesis, will require being treated according to the indications for the cure of the latter affection, which will be hereafter considered.

7°. Irritation of the lungs tending to the production of phthisis, provoked by suppressed cutaneous affections, hæmorrhages, and inflammations, should generally be combatted by the antiphlogistic regimen, aided by other measures adapted to the particular indications of the individual case.

The irritation of the mucous membrane of the intestinal canal, tending to the production of tabes mesenterica, must be treated according to the principles already developed in some of the preceding articles.

The inflammations which appear in subjects affected with scrofula, are produced by the same causes, and should be treated after the same principles, as those occurring in persons in general; only bearing in mind the disposition to activity of the lymphatic vessels, and relative want of vigour of the sanguiferous system, which should lead to a more reserved use of active antiphlogistics than in persons of the opposite disposition, and to the employment of revulsives as soon as the subsidence of the more severe degree of the inflammation will permit. It is an error to suppose, because tonics tend to remove the disposition to scrofula, that they are appropriate for the treatment of irritation of the sanguiferous system which may accompany, or be consequent on, the existence of that disposition.

In a former part of this exposition (vol. xli. p. 243,) we stated that "when the nutritive materials are insufficient in quantity, or are to a certain degree improper for nutrition, the animal economy falls into a state of debility, which often proceeds to actual disease;" that "other agents, such as cold and humid air, painful moral affections, &c. frequently debilitate the system, without producing previous irritation;" and that "the continued use of innutritious excitants will produce local irritation, at the same time that they influence the rest of the system in the manner just indicated." These principles point out the origin and nature of scurvy. Thus, it is observed as a consequence of the long-continued use of salted, smoked, and dried, aliments, as the principal food, whatever may be the qualities of the air, water, and other circumstances, surrounding the patients of that disease. Those materials, so ill appropriate for the alimentation of the body, although at first only leading to a decomposition of our organs from want of nutrition, do not always confine their effects to this; they often produce an irritation of the whole lining of the alimentary canal that passes at length to inflammation and disorganization; and at length the same effects ensue in the serous membranes, in the muscles, in the articular capsules, and even in the extremities of the spongy bones. Thus, we find in the dead bodies of scorbutic patients, phlegmasiæ of all species, with morbid adhesions, sanguineous and serous effusions, depositions of matter and gangrene in the parenchyma of the liver and lungs; the cavities of the joints filled with fluids of different kinds, and their capsules thickened and disorganized; the cartilages and heads of the bones carious and friable, the epiphyses separated and floating in sanious fluids; the muscles not only gorged with blood, hard, and infiltrated, but even reduced to a sort of clotted, half-putrid, mass, sometimes exactly filling their aponeurotic sheaths; sometimes the muscular layers are shrivelled, and contain pus similar to that of phlegmonous abscesses. But, in the midst of all these disorganizations, the brain and rest of the nervous system appear to be unaffected. This, as we shall presently show, will explain one very remarkable circumstance characteristic of this disease. Let us first trace the general progress of the phenomena by which it is constituted. The species of food which we stated to give rise to scurvy, fails, in the first instance, to supply to the blood the necessary quantity of nutritious materials: the muscles, which, in the healthy state of that fluid, appropriate to themselves the fibrine it contains, no longer obtaining from it the substance necessary for their action, experience the first effects in this disease. Their contractile power is gradually weakened, and is at length totally lost: thence the langour of the patients, the extreme fatigue they suffer from the slightest movements, and at last their absolute impossibility to move their limbs. The heart participates in this debility of the exterior muscles: it no longer propels the blood with any considerable force through the arteries; that fluid stagnates in its cavities, in the large veins, and successively in all parts of the body. The weakness of the heart is so great in some cases, that it becomes distended by the blood with which it is surcharged, and is found in the dead body dilated to a very considerable extent: and, even in those patients who recover, it remains particularly liable to aneurismal dilatation. Absorption is then necessarily obstructed; collections of serous fluids take place in the extremities, and in the different visceral cavities. Thus, the first morbid effects in scurvy are of an organic nature, or, in other words, exist in the change in the properties of the solids; but a depraved state of the fluids soon ensues; the blood loses its proper constitution, and the secretory organs, languishing in their functions, give rise to an accumulation of effete matter in the vessels, which irritates the organs with which it is in contact, and produces at length the inflammations in the different parts which have been pointed out. It should be remembered, too, that the subjects are liable to have inflammation excited by all the common external causes of that affection, as well as by the peculiar internal ones that have just been designated.

"Represent to yourself," says M. Broussais, "the capillaries and the muscles of scorbutics in that state of weakness which depends on the want of adhesion between their molecules: what should be the result of the precipitated vibrations and of the engorgement which the phlegmasiæ determine in them with the more violence, because the nervous system, the only cause of organic movements, is perfectly healthy in this affection?—a very ready passage to gangrene, a rapid disorganization." The occurrence of such intense inflammation in patients affected with scurvy, has been brought forward as an argument in favour of the origin of inflammation from local debility,—that is, that debility of the vessels immediately give rise to it; but the above observations will show on what improper grounds. The hæmorrhages which take place in scurvy may, according to the opinion of M. Broussais, be of two species: the one similar to acute hæmorrhages in general, from irritation of the sanguineous capillaries; the other from disorganization of those vessels. The ecchymoses, as they generally appear, probably depend on the obstruction to the progress of the blood in the veins. Although it is stated in the foregoing account, that the alteration in the properties of the blood gives rise to inflammation of various parts in scurvy, yet this is by no means a frequent occurrence; and it is never witnessed except in very prolonged cases of the disease, except the patient makes use of alcoholic fluids, spices, or other powerfully-irritating matters; and these probably effect the inflammation and disorganizations alluded to, by first producing irritation of the gastric organs, whence it is communicated to the rest of the system. We find persons suffer scurvy for several months, who use a sort of diet not calculated to produce irritation of the stomach, who have become reduced to the utmost state of debility, without those disorganizations taking place; but if, from any accidental cause, inflammation of the stomach is induced, they immediately follow, and terminate the life of the patient without delay: and dissection shows that the disorder may terminate fatally without the disorganizations resulting from inflammation having taken place. This explains how persons bear deprivation of food to a great extent, for a long period, without becoming affected with the phenomena characteristic of scurvy: to produce these, the aliment must possess more or less irritating qualities; and, in proportion to the degree of excitement of the gastric organs, will be the severity of the characters of this disease.

The nature of scurvy, then, according to the doctrine of M. Broussais, consists in, 1st. Alteration of the blood, in consequence of want of proper nutrition; 2d, irritation of the mucous membranes of the gastric organs; 3d, weakness, and at length the loss, of muscular contractility; 4th, obstruction to the circulation of the blood from debility of the heart; 5th, irritation more or less considerable of the sanguineous capillary vessels in all the organs, extravasation of blood, destruction of different parts; and, in the midst of this extensive disorder, integrity of the texture and functions of the nervous system.

The treatment of scurvy is so clearly pointed out by the foregoing theory, that it is not necessary to say much on this subject; it is the same, according to this doctrine, as that which all judicious physicians have stated to be most beneficial; fresh vegetables and acidulous fruits in the first instance, and then a more liberal supply of nutritious food. The above doctrine, however, furnishes an indication against the use of stimulants that has sometimes been deviated from; but always with injury when employed before the state of irritation of the stomach and small intestines has been removed. "What a lesson for the Brunonians does this disease afford, (exclaims M. Broussais,) who personify the results of our functions, and who have only two confused ideas, according to which they pretend to explain all the phenomena of the living body!"

---

*History of the Petechial Fever of Genoa in the years* 1808 *and* 1809. By G. Rasori, M.D. &c. 8vo. Milan, 1813.

From the facts related in this work another theorem may be deduced, not less important than that already proposed: it regards the mode of operation of some substances, which the author employed with so much efficacy in the treatment of the petechial fever; and to which, in conformity to the general language, we have given the name of *anti-phlogistics*. Let us remember that those substances were principally tartar-emetic; mineral kermes; the common supertartrate of potash; and some saline purgatives and diaphoretics, as they are usually termed; now, we may reduce to two the opinions which, in 1800, were most generally prevalent amongst physicians respecting the medicinal qualities of those saline substances. One was the opinion of the followers of Brown, and that most generally adopted. The Brunonians considered those we have mentioned in the aspect of stimulants; and, amongst the most active of stimulants, they placed the kermes; and they attributed so much efficacy to it, that, fortunately, they did not hesitate to employ it even in that peripneumony, which they chose to term asthenic; they also considered the tartar-emetic, and other substances above enumerated, as incitants: that is, if they did not, as frequently happened by means of the immoderate evacuation they produced, become indirect sedatives, or rather debilitants. So, whenever subtraction of the fluids from the body did not arise from their action, their effects were considered as stimulant; and, by this happy error, those medicines sometimes were employed with success in certain pretended asthenic diseases. On the contrary, those entertaining the more ancient notion, never acknowledged their general properties, which Brown first investigated with a philosophical spirit, and discovered through so many varied forms in different medicines; they did not consider these substances with the view to discern whether they principally augmented or lessened the vital power, or as they have since been more philosophically regarded, whether they produce excitement, or an opposite state. It was the last and particular, or in a manner local, effect that might be expected from them which they solely took into consideration. Thus, they saw in the kermes an *expectorant* or an *incisive;* in tartarized antimony, an *emetic* or a *sudorific;* in the cremor tartar, a *purgative* or a *diuretic:* and all at the best agreed in classing, in a general manner, some of the remedies of that kind under the common denomination of *anti-phlogistics;* meaning to say that the various *crises,* effected by their means, tended to destroy the phlogosis or state of over-excitement; and it should be remarked, that those good old practitioners relied so firmly on the idea of the especial production of this crisis or evacuation, that none of them believed the substances designated ever acted in a beneficial manner, unless some critical evacuation of the *peccant humors,* or of *saburræ,* or of the *morbid fomes,* followed their use.

Such were the opinions of the medical world, new and ancient, when the fortunate experience of Rasori, in the epidemy of Genoa, came to open the eyes of physicians. 1st. Many of the patients were treated with those substances. 2d. They were administered in such doses, that, if the remedy were appropriate, they were sufficient to prove beneficial; whilst, if they had been inappropriate and contra-indicated, they could not fail being injurious. 3d. Their sensible and ultimate effects were the permanent cure of the disease; which necessarily indicates an appropriate method of treatment. 4th. The use of those substances was, in some cases, accompanied with proportionate evacuations. But, 5th, in a much greater number of cases, no evacuation was manifest, or not manifest in a degree at all appropriate to either the intensity of the disease, the degree of amendment, or the extent of the dose: thus, the most remarkable phenomenon, observed from the action of those remedies, was the restitution of health; and their evacuating effects, generally speaking, were less in proportion to the greater intensity of the disease. Let us then reason dispassionately on those data, relinquishing all prejudice and favourite maxims, to listen to, and profit by, the useful lessons they furnish.

We then thus address the disciples of Brown;—In what way will you explain the operation of the tartar-emetic, kermes, nitre, and supertrate of potash, in those cases; and the mode in which they effected the cure? By stimulating? That is not possible. Reconsider the numerous arguments by which it has been shown that the fever of Genoa, even in the period called nervous, preserved its inflammatory diathesis. Can it be said that an inflammatory disease may be cured by stimulants? Consider that when these substances were beneficial, opium, wine, and the true stimulants, were manifestly deleterious. Is not that an argument that the one operated in a manner contrary to the other? Reflect that the same salutary effects which were obtained from the tartarized antimony, and other saline substances, were also obtained from general blood-letting, and from scarification, which certainly are sedative. Do not similar effects forcibly indicate a similarity of causes? But, do you require an argument that cannot be replied to? The medicines above alluded to, in many cases, produced an easy, decisive, and rapid cure: in others they produced the best effects, in too great a number of cases to permit their success to be attributed to hazard, or proportionate evacuation, after the occurrence of which you are ready to acknowledge that the stimulant action is very far inferior to the debility induced by the subtraction of the fluids. They then cured, although they so much debilitated? Then they cured, because they debilitated. Can it in reality be believed that, unless that were the case, the return of health would have been effected, in such a state of disease, in a manner so manifestly connected with the use of those remedies, so rapidly and so undisturbed by any sinister accident.

You are then necessarily led to admit, that the re-establishment of health, in the former cases produced by a stimulant action, was in the latter produced by an opposite mode of action: but will you now say, that if those remedies manifested a debilitant curative action, that it was indirect debility, resulting as a consequence of the evacuation they produced, without which their action would have been purely stimulant? It will be easy to show the erroneousness of your reasoning. Although it has been allowed that proportionate evacuations were not wanting in many cases, yet such evacuations were by no means present to such an extent as was expected in a much greater number of others, which, except in this respect, were identical with the former. There were the same nervous characters present in these as in the former. In both, the use of opium, ether, and alcohol, was so manifestly and promptly injurious as not to be at all equivocal. On the contrary, the remedies that were beneficial in the one, were equally so in the other, and in the same stage of the disease. Those remedies also showed themselves equally salutary, whether they did or did not produce evacuations: independently of the presence or absence of these their effects were directly opposite to those resulting from substances decisively stimulant. The only consequence to which we are led, then, is this: *the tartar-emetic, and other saline substances above enumerated, directly diminish excitement, although no evacuations are produced.*

You are wrong, then, Brunonians! in asserting that all medicines stimulate: there are some which manifest a contrary action to that of stimulants, immediately and without any abstraction of fluids. There are then *contra-stimulant* powers; and this is the second grand truth which Rasori has the merit of having developed. In the work on the epidemy of Genoa we only find the first foundation of this truth. We shall hereafter have to notice some further arguments by which it is rendered more evident. Those already advanced have peremptorily established it; and many have not been noticed that might have been adduced. We might, for example, show that the small quantity of water or of gastric fluid evacuated from the stomach by the action of tartar-emetic, does not bear any relation in degree to the great faintness and weakness which it often produces. We might add to this, that the antiphlogistic effects of the nitre were too considerable to be attributed to the small increase of the quantity of urine or of sweat which it produced. To these and some other similar arguments we shall return at a future period. Here we terminate our dispute with the disciples of the physician of Edinburgh: the followers of the more ancient school will now engage a little of our attention.

The greater part of these certainly insisted much on the importance of attending to the symptoms of diseases; but they never raised their views of the nature of remedies to their general stimulant or contra-stimulant qualities: thus, they would pretend, that, if cremor tartar, tartarized antimony, nitre, and mineral kermes, were beneficial in the petechial fever just described, and in many other analogous disorders, they were beneficial only in this manner; that the one, for example, would act as an *incisive* and *resolvant* of the ill-concocted matter which, being thrown on the lungs, had oppressed them; that the other would dissipate the *saburres* of the stomach and intestines, either by stool or vomiting; the third would relieve by the urinary passages, or by the pores of the skin, the sanguineous system of the morbid matter, when in the torrent of the circulation it is transported into their circle. —But there need no other means for the destruction of this venerable system, than the clear evidence of facts. No doctrine is more efficaciously combatted by the simple history of the epidemy of Genoa, than the doctrine of the symptomatic physicians. What rules were observed, then, by Rasori? It seems that he took, as it were, a pleasure in applying those remedies in a sense expressly contrary to those maxims, being secure of his favourable success. Would he, in conformity to the indications of the symptoms, when he saw subsultus of the tendons, lethargic stupor, and placid delirium, have resorted to tartarized antimony, and not rather to musk and the anodyne liquor? Would he have had recourse to purgatives, or to emetics, in the advanced stage of the disease,—in the malignant period, when there was no meteorism, nor indications of gastric disorder, present? Would he have used kermes, when the chest was free? or nitre, when the urine was copious? Would he have terminated the cure with emetics, diuretics, purgatives, leeches, and scarifications, without sinapisms, without blisters, without serpentaria, without ammonia? Would he have administered ice, when there was danger of phlogistic re-action? And, above all, would he have placed the least confidence in saline medicines and in tartar emetic, without producing any evacuations by fæces, vomiting, copious flow of urine, or profuse sweating, according to the pathognomonic indications of the *materia morbosa?*

Rasori, then, treated the disease in a very different manner than from the indications of the symptoms. He entirely neglected all those that are commonly considered as the most dangerous; and, even though the remedies he adopted might, according to the old doctrine, be theoretically reduced to symptomatic medicine; in the greater number of particular cases where he adopted those remedies, they were either contra-indicated according to that doctrine, or they should not have been beneficial, because they did not produce the effects that were expected from them.

But, if Rasori did not treat the symptoms, and nevertheless produced a restoration of health; if his remedies were not conformable to the indications of the symptoms in themselves; on what principles, then, did his remedies act? Not as emetics, diuretics, and cathartics; because they were so frequently beneficial without producing vomiting, diuresis, or catharsis. Not as antiphlogistics, in the old sense of that term; since they were beneficial in many cases where no evacuations were produced, (even when there did not exist the characteristics of clearly and well-marked inflammation,) and where no sensible crisis ensued. How, then, we repeat, were those remedies beneficial?

Disciples of the various schools anterior to Brownism! you cannot refuse to admit that those substances possess other qualities than those of purgatives, emetics, and sudorifics; without the exertion of which we have seen them effect the restoration of health: you must allow them other qualities than the incisive, the attenuant, the eliminatory of the morbid matter, &c. since we have been shown such an hypothesis to have been inadmissible in a great number of cases: you must admit in them a third general quality, to which a too unimportant, and merely secondary, character has been attributed; we mean a refrigerant, antiphlogistic one, or, in other words, a sedative of excitement. You must perceive that those remedies, or those principles in general, had an effect directly opposite to that either of wine, of opium, of ether, or of alcohol; and you so well discern it, that you fear to employ them to a considerable extent where there exists great real or apparent debility. You, then, confess that they destroy phlogosis where it exists; and moderate vital excitement, already, as we say, carried to an inordinate extent. In this respect you are in the right; but you must not too far subtilize your reasonings on this point, and attribute the effects above designated to the evacuations they produce. Experience in the petechial fever of Genoa, supported on other occasions, has demonstrated, and in such a manner as not to admit of reply, (by facts), that those effects were produced without any evacuations. If we then isolate from what is proved all those circumstances which are not constant and well-ascertained, and which it is useless to enter into the consideration of, we shall then feel assured that the remedies in question must have produced their effects on different principles from those which you assume. In the fever of Genoa, the medicines used certainly could not operate by stimulating, since they caused no evacuations: they did not operate by an elective faculty, or as cathartics, emetics, expectorants, diuretics, or diaphoretics: they did not act by dissolving or attenuating lentor of the fluids, or by producing decomposition of the morbid matter. We then first learned, from observation worthy of the age in which we exist, that they produced their beneficial effects by an agency directly opposite to that of a stimulus. There exist, then, medicinal substances, which, contrary to the doctrine of Brown, cause sedative effects independently of any subtraction, and therefore what may be properly termed *contra-stimulant* agents, as it has been thought proper to term them, (we will not now dispute on the propriety of names;) and this we consider to be one of the greatest of the many truths disclosed in the work of Rasori, and one which we shall find to have been confirmed by a long series of subsequent observations.

The existence of sedative substances was long since known; but the sedatives of the ancients are not the contra-stimulants of Rasori. Opium was a sedative with them,—if not castor! They not only considered as sedatives those substances which immediately diminished action, but those also which depressed excitement by first causing inordinate action. Opiates and sulphate of soda were used at the same time as sedatives. They looked to the ultimate effects without considering how they were produced, and were thus constrained to arrange under the same class all the remedies of the materia medica, since all of them are in some cases sedatives on their principles; and thus to comprise, under the same title, substances of the most opposite nature.

Antiphlogistics were also known in former times; but the antiphlogistics of the ancients present too circumscribed, and indeed false, an idea of the remedies which solely oppose phlogosis; and the contra-stimulants of Rasori are more than the antiphlogistics: they combat, in general, all states of excitement at all accompanied with the inflammatory process. Besides, the antiphlogistics of the ancients were only those acid, or sub-acid, mineral, saline, substances which refrigerate the palate; whilst there are many contra-stimulants, according to Rasori, that do not produce that effect.

The Brunonians had their sedatives; but the sedatives of the Brunonians, besides being thought to act thus always by means of subtraction, or as negative stimulants, presented to them another erroneous idea; since they gave rise to an idea of principles, which induced, by means of their action, a state of debility or of lessened excitement. When it has happened, then, that a disease of an inflammatory nature has been accompanied by the phenomena of depressed excitement, as was the case in the fever of Genoa, the use of those sedatives, as they are termed, has not unfrequently realized their ideas: depressed excitement being evinced by the occurrence of death. It is not, then, a renewal of an old maxim, but the discovery of a new truth, or, at least, the rectification of some erroneous views of the ancients, that we owe to the Clinical School of Milan.

### *Some Considerations on the Pupillary Membrane; on the Nature of the Liquid contained in the two Chambers of the Eye, &c.* By M. Portal.*

The ancients had no knowledge of the pupillary membrane,—of that membrane which completely fills up the opening in the iris termed the pupil. Riolan clearly showed, towards the end of the fifteenth century, that infants do not see at the time of birth;† but he did not attribute the cause to the existence of the pupillary membrane, of which he does not speak.

Littre also stated, at the Academy of Sciences, in 1707, that blindness at the time of birth was produced by a membrane which obliterated the pupil; but he did not give any description of it. It appears that this anatomist considered this membrane as an extraordinary or morbid formation.

Wachendorf, a German physician, is the first that made it known through the medium of the press, in 1740.‡ Two years after, Haller, who was not acquainted with the dissertation of Wachendorf, gave a more exact description of it, with a figure,§ believing himself to be the first who had observed this membrane; but he soon afterwards became informed of the observations of Wachendorf, as he states in his great work on Physiology.‖ It is very remarkable, that Albinus, the immortal master of these two anatomists, claimed this discovery, in 1756, in his *Annotationes Anatomicæ*, asserting that he had discerned it as early as 1737. But, as neither Wachendorf nor Haller had heard Albinus speak of it, they cannot be blamed for not having recognized him as the author of the discovery.

On examining the eyes of fœtuses who had died in the uterus, Haller perceived, in the aqueous humour, an apparently floating plexus of vessels; but, as he knew that vessels are sustained by membranes of some kind, he suspected that the pupil might be obstructed by such a texture. This suspicion led him to make a series of researches, and he became convinced that the pupil of those fœtuses which he dissected was closed by a rather strong white membrane, in which were dispersed several vessels extending into it from the iris. This membrane, which appeared white to Haller, closes the pupil so exactly, he says, that it prevents the rest of the aqueous humour flowing away, when that in the anterior chamber has been evacuated by an incision in the cornea.

Although the description of Haller left but little to be desired, yet the subject still engaged the attention of several anatomists, and particularly of Zinn, who not only more fully described the membrane

* Taken from the fourth volume of his *Mémoires sur la Nature et le Traitement de plusieurs Maladies*. 8vo. pp. 358. Paris, 1819.

† *Anthropographia*, p. 409.

‡ *Comment. Literar.* Nuremberg, 1740.

§ *De Membrana Pupillaria;* in the works of the Society of Upsal, 1742.

‖ *Element. Physiol.* tom. v. p. 372.

under consideration, but indicated, also, the mode in which it might be clearly seen and demonstrated.*

Other celebrated anatomists, amongst whom were Hunter and Blumenbach, also recognized and demonstrated the pupillary membrane: Wrisberg, especially, has given a very detailed description of it, in his *Commentationes Medicæ.*†

The pupillary membrane is not ordinarily found in infants who have lived for a short time after birth, which has led me to believe, after Gataker, a celebrated German anatomist, and Haller, that this membrane, if it has not already been ruptured by causes which are unknown to us, (which does sometimes happen, because it cannot always be found,) is broken during, or a short time after, the birth, either by the effects of the contractions of the muscles of the globe of the eye, when the infant has come into the world, or in consequence of the abundant secretion of the aqueous humour of the two chambers which takes places after birth; which, enlarging them, must necessarily produce an extension of the iris, whence the rupture of the pupillary membrane may arise. This increase of the aqueous humour is the more remarkable, because the transparent cornea of the fœtus which has not respired, is generally less prominent than that of infants who have lived; and, besides that, the aqueous humour is then opaque, thick, of a deep colour, or more or less black; whilst, on the contrary, it is more limpid and clear in the infant who has respired for a certain length of time. It appears that this humour loses its opacity in proportion as it is mingled with the new aqueous humour which flows into the two chambers of the eye after birth. May we not also believe, that the impression of light on the retina, which produces contractions of the iris in the whole period of life, may excite powerful ones in the newly-born infant, and that a rupture of the membrane may thence result?‡ I have been convinced, on examining the eyes of some infants who have lived one or two days, that there remained, about the opening of the pupil, some little fragments of this membrane, which disappear ordinarily more or less rapidly; which is much less surprising than the annihilation of several other parts of the fœtus, which are no longer found in the course of life, as I have stated, when speaking of the annihilation of the crystalline, altered by chirurgical operations, or other causes, in a memoir on this subject.§

The pupillary membrane is not, however, always ruptured at the time of birth. It has several times been recognized, in its state of integrity, six or eight days after, and even later.

Anel was persuaded, from the results of his own observations, that this rupture did not take place until towards the seventh week after birth.

---

† Göttingen, 1800.

‡ This supposition of M. Portal is not very well founded, because *contractions* of the iris must rather cause a more lax state of the pupillary membrane than a distension of it.

§ *Annales du Muséum d'Histoire Naturelle*, 1805, tome ii.

We have stated that Littre believed that the blindness which continues during life, might be an effect of its presence.* I also stated, in my *Anatomie Medicale*, that, if the rupture of the pupillary membrane does not take place, children remain blind. Such has doubtless been the cause of blindness from the time of birth, of which some oculists have spoken: without well understanding this cause, they have advised the perforation of the iris, to make a sort of pupil; not to open that which was obliterated by the pupillary membrane, with which they were not acquainted, but in other parts of the iris, more or less near the place where the pupil should appear, in order to effect it as nearly as possible in the axis of vision;—an operation which has been successfully performed, both to remove this cause existing from the birth, and others which subsequently give rise to obliteration of the pupil.

The pupillary membrane, which is of a circular form, is not, at most, more than two or three lines in diameter; the pupil being, in the fœtus not yet entered into the world, in its state of greatest dilatation. On examining it closely with the microscope, we see that it intimately adheres to the interior margin of the circumference of the *pupil*:† it appears smooth and uniform on its surfaces corresponding to both chambers of the eye, and so thin in some places, especially in its centre, that it seems transparent.‡ When it is examined before the light,§ several more or less opake, tortuous, lines are seen, many of which are extended in the manner of rays; they are formed by the ramicles of vessels which communicate with those of the iris.

This membrane is formed of cellular tissue, in a more or less condensed state. I have not been able to divide it into layers. This *caduceous* membrane (if I may be allowed so to term it) is incomparably thinner than the iris, in which we may observe, 1°, a portion of the membrane which lines the anterior chamber; 2°, the anterior proper layer of the iris, intersected by vessels differing in colour; 3°, the posterior membrane of the iris, to which the name of *uvea* has been given, from the darkness of its colour; 4°, a part of the hyaloid membrane which lines the posterior chamber.

All these membranes, united by a common cellular tissue, in giving more thickness to the iris, essentially distinguish the structure of it from that of the pupillary membrane, which is, as I have stated, thinner, more fine, and very easily torn. I should here add, that it is from the hyaloid membrane, which lines in an uninterrupted manner the two chambers, comprising the two surfaces of the iris and the mar-

---

* Wrisberg discovered that this membrane was entire in both eyes of an infant born blind, and continued so to the age of three years and a half, when it died of the small-pox.—See his *Comment. Med.* Göttingen, 1800.

† Of the *iris*, it should be said; the *pupil* is a mere non-entity,—a term used to designate the space surrounded by the iris. This error can only have arisen from an inadvertency of expression, in such a man as Portal.—Edit.

‡ This vicious mode of expression should be abandoned: it often gives rise to confusion in the ideas language should convey. A body in itself cannot be *transparent:* it may admit of other bodies being *trans parent.*—Edit.

§ Wrisberg also remarked, that this membrane was extremely thin, and composed of many vessels coming from the iris: it varies, however, in a remarkable manner, both with respect to its aspect and its structure.

gin of the pupillary foramen, that the cellular tissue is derived which gives rise to the pupillary membrane.

Let me add to this account of the pupillary membrane, the existence of which may prevent infants seeing at the time of birth, that I am equally persuaded that they do not hear on coming into the world, any more than see, and even that such is the case more generally. I form this opinion from the circumstances which I am about to detail.

It is certain that there are many cases of deafness which may arise from the cavity of the tympanum being full of mucous or other humours. The results of anatomical and pathological observation have so frequently proved it, that we cannot doubt of the existence of this species of deafness. Now, as in the fœtus, the cavity of the tympanum is always full of mucous matter, as well as the Eustachian tube, of which I have been several times convinced by an attentive examination of the internal parts of the ear, I do not doubt that this abundant existence of mucus may essentially cause deafness, which may continue as long as the cavities above designated remain full of it. It appears that it is only when the fœtus has respired, and that the pituitary membrane has become freed from the mucous matters with which it is covered, and which fills the nasal cavities, as well as the Eustachian tubes and the tympanal cavities, that infants begin to hear.

Indeed, when we examine the ears of those who have lived a few hours, we find them no longer equally filled with the mucous matters: this I have several times ascertained. I have no doubt but that children remain deaf and dumb from their birth from this cause, if it be permanent, or because the cavity of the tympanum remains incommoded by some other matter. At other times there exists a want of the development of the auricular cavities,—an organic vice which I have found in one case of primeval deafness.

---

*On Mediate Auscultation; or, a Treatise on the Diagnosis of the Diseases of the Heart and Lungs, principally founded on this new Mean for Exploration.* By R. T. H. Laennec, Doctor in Medicine of the Faculty of Paris; Physician to Necker's Hospital, Honorary Physician to the Dispensaries, Member of the Society of the Faculty of Medicine of Paris, and of several other National and Foreign Societies. 2 vols. 8vo. of xlviii. and 456, and 472, pp. *Brosson,* Paris, 1819:* with the epigraph,

Μέλα δέ μέρος ἡγεῦμαι τῆς

Τέχνης τὸ δύνασθαι σκοπεῖν. Hippoc. *Epidem.* iii.

By the expression *mediate auscultation*, the author intends to designate the attention of the mind to the phenomena of diseases perceived by the sense of hearing, through the medium of an instrument: a mode of exploring maladies, when applied to the thoracic organs, somewhat analogous to the application of the ear simply to the chest, recommended by Hippocrates, and the manual percussion so much

* *De l'Auscultation Médiate; ou Traite du Diagnostic des Maladies des Poumons et du Cœur, fondé principalement sur ce nouveau moyen d'Exploration.* Par R. T. H. Laennec, d.m.p. &c. &c.

favoured by Avenbrugger; but so far superior to them, that it furnishes, for the most part, "*novel, sure, and striking signs, easily to be discerned, and calculated to render the diagnosis of almost all the diseases of the lungs, the pleura, and the heart, more certain, and perhaps more circumstantiate, than surgical diagnostics established by the aid of the sound or the touch of the finger.*"

A wooden cylinder, a foot long, from eighteen to twenty lines in diameter, divisible in the middle of its length by a screw, (to render it conveniently portable,) pierced in the centre by a tube nine lines in circumference; but terminating at one end by a tunnel-like cavity, about an inch and a half in longitudinal dimension, and of nearly an equal extent in diameter at its large extremity, with a sort of plug, itself pierced in the middle by a canal three lines in diameter, to be occasionally inserted into this tunnel, by which it is rendered a simple cylinder,—is the instrument used by M. Laennec, and to which he has applied the term *stethoscope.*

Here we stop in our description, to have a little personal converse with the reader, in order to remove the prejudicial and incorrect sentiments he will probably form respecting the present subject of our labours. Two large volumes devoted to the diagnosis of the diseases of the thoracic organs, and by means of the signs perceived by one of the senses only, by the aid of an instrument too, in which, without a jaundiced eye, a little charlatanism might be suspected, are certainly not good auspices. We ourselves formed similar ideas; but we had not perused many pages of the work before we began to blame the author for having given to it the title it bears; and, on proceeding a little further, we felt disposed to censure the method he has chosen of making *the principal object of his work the designation of the advantage that may be derived from the use of the cylinder in distinguishing the different lesions of the lungs, and rendering every thing else subordinate to this design: the facts in pathological anatomy, even although occupying a greater extent of space, being inserted merely as accessories.* We regard these facts, many of which are novel and of the most interesting kind, as forming a part of the work certainly not *inferior* in importance to the new means of diagnosis; although we are not one of those to whom a passage in the dedication* is, in this case at least, applicable: *Nostra enim ætas incuriosa quoque suorum; et si quid novi ab homine coevo in medio ponitur, risu ut plurimum ineptisque cavillationibus excipiunt: quippe facilius est aspernari quàm experiri.*

Besides the novel pathological observations above alluded to, this work contains extensive judicial remarks on the value of the signs of the diseases of the thoracic organs; and some important indications for their cure, especially for that of phthisis. This part constitutes an excellent specimen of the results of the philosophic spirit with which pathology is studied in France at the present period. We know not how to speak more forcibly in its praise.

We shall, then, take a somewhat different course from that which

* To the College of Physicians of Paris.

Laennec has done in the exposition we are about to give of the contents of his work. Pathology shall engage our first and chief attention; and we shall treat of the new means of diagnosis in a secondary way, as it is treated of in medical writings in general; taking care, however, to omit nothing of considerable importance respecting it.

In order to open as clear a view as possible before the reader, and to favour conciseness of narration in our progress, we shall commence with a general account of the phenomena developed by the stethoscope, in the state of health especially, and of the mode of exploring them. Those phenomena relate to the *voice*, *respiration*, *rattles*,* and the *action of the heart*. The instrument should be held as a pen is done, and applied to the chest steadily and firmly, with the hand near to it, so as to secure its proper application. The end of the cylinder, in which is the tunnel-like cavity, is to be in contact with the patient, and the orifice of the ear of the physician gently pressed against the opposite extremity. When the voice and the movements of the heart are explored, the perforated plug is to be inserted. In extremely meagre persons, the surface of whose chest presents grooves between the ridges of the muscles and bones, some cotton covered by a piece of cloth, or a piece of soft paper folded, should be placed beneath the end of the stethoscope. This will often be requisite on the sternum. We need not treat of the manner, and peculiar force, with which sound is conveyed through long rods of hard and dry wood, and simple tubes: these things are well known by every person the least acquainted with the science of physics.

1. *On the exploration of the voice.*—When a healthy man speaks or sings, his voice resounds through the interior of his chest, and produces throughout the whole of the parietes of this cavity a sort of thrilling which may be easily distinguished by the hand, and which is very forcibly perceived by the ear by the aid of the stethoscope. But when, by the effect of disease, a lung has ceased to be permeable to air, this phenomenon ceases in the corresponding side of the chest: the same thing occurs when there is a considerable collection of fluid in the chest, although the lung be sound. It is not very sensible, also, in extremely fat persons, whose integuments have a certain degree of flaccidity, and in those whose voice is very shrill-toned and weak. No important indication can, however, be derived from this phenomenon, because it is not possible to determine the precise extent of the lung which is, or is not, permeable to the air. It may however be concluded, when this vibration exists, that a great portion of the lung is permeable. The hand, too, furnishes better evidence on this point than the stethoscope; and it is proper to add here, that the two means should, in all cases of exploration, be conjoined, as the one may furnish evidence in support of that acquired by the other.

When a cavity of any considerable extent exists in the lungs, a very

* By *rattles*, M. Laennec signifies all the sounds produced by the passage of air through liquids contained in the bronchiæ or pulmonary tissue. We hear the term commonly used in England, by nurses, to designate that which occurs in dying people only, as it is only in this case that it can be heard by the ear simply. This will be presently explained.

curious phenomenon may be detected by the stethoscope; for, on applying it to the parietes of the chest, corresponding to this cavity, the voice of the patient seems to come through the cylinder, and this more forcibly in proportion as the cavity is nearer to the surface of the affected lung. This phenomenon M. Laennec terms *pectoriloquism*. It is precisely similar to what is heard on applying the stethoscope to the trachea in a healthy state. The parts of the chest where it is most frequently heard, are the anterior and superior portion, the axilla, the space between the clavicle and the trapezius muscle, and the fossa above and below the spinous process of the scapula. It may be perfect, imperfect, or doubtful. When it is very perfect, it is a certain sign of the existence of a preternatural cavity in the lungs. It is more clearly evident in proportion to the acuteness of the voice of the patient; when the voice is grave or hoarse, the pectoriloquism is like the voice coming through a speaking-trumpet or roll of paper. This is also perceived in cases where the cavities of the lungs are very large in size. In some cases, it seems to traverse a brazen tube with a remarkable and characteristic sort of bleating (*chevrottement*) sound: this is *egophony*. In others, at every word the patient pronounces, there is a tone distinct from that of the voice simply, a ringing analogous to that of a little bell or a glass that is just ceasing to sound, and which seems to rise to a certain height in the cylinder, and then die away: this is *metallic ringing*. The causes of these varieties of pectoriloquism will be hereafter explained, and many other peculiarities described, not appropriate for notice in this general view.

2. *Exploration of the respiration.*—When the stethoscope, used with the tunnel-like cavity, is applied on the chest of a healthy man, a gentle, but very distinct, murmur is heard, which indicates the passage of the air into the air-cells of the lungs, and its expulsion thence. The axilla, and the space between the clavicle and the trapezius muscle, are the parts where it is most forcible; but it may be discerned all over the parietes of the chest. It is heard very well over the larynx and the cervical portion of the trachea; and even, in many men, throughout the whole extent of this canal: but, on the trachea, and even to a little distance on the roots of the bronchiæ, the respiration has a peculiar character: the air seems to pass into a canal which is larger than the pulmonary cells; it seems sometimes as if the air were drawn from the cylinder, and passed through this canal. The thickness of the clothes diminishes it but little; the same remark will apply to fatness, and infiltration of fluid in the thoracic cavity. Respiration is more sonorous in proportion to its frequency. In infants it is very sonorous, and it has in this age a peculiar character, which is simulated in some diseases. When it is heard distinctly and nearly equally throughout the whole chest, there is neither infiltration nor engorgement of the lungs. When it is not heard in any point, the corresponding part of the lung is impermeable to the air.

3. *Exploration of the rattles.*—There are four principal species of rattles: 1°, the humid rattles, or *crepitation*; 2°, the mucous rattles, or *gurgling*; 3°, the dry, sonorous rattles, or snoring (tracheal); 4°, the dry, hissing rattles, or whistling. The first takes place in the first stage of pneumonia, in œdema of the lungs, and sometimes in hæmoptysis. The sound is very similar to that heard on pressing a portion of a healthy lung between the fingers. The second are produced by the passage of air through collections of mucous or similar matter accumulated in the bronchiæ, or through softened tuberculous matter in a cavity in the lungs. They are the rattles of dying persons. These alone are heard by the ear simply, and then only when they have their seat in the trachea or large ramifications of the bronchiæ. By the aid of the stethoscope, they may be heard, as well as the other species, in all parts of the lungs. The sonorous rattles present more variable characters than the two former species. They consist in sounds more or less grave, which are sometimes similar to the snoring of a sleeping man, sometimes to the sound rendered by a bass-cord when it is struck by the finger, and very often to the cooing of the turtle-dove. The last variety only takes place, in general, in a small portion of the lungs. It often arises from pulmonary fistulas of moderate capacity, and occasionally from dilatation of the bronchial tubes.* The sonorous rattles must not be confounded with guttural snoring, which arises from a peculiar mode of impression of the inspired and expired air on the velum of the palate. The ear simply might confound them, but the cylinder will lead to a detection of the difference. The hissing, or whistling, rattles, may be either prolonged, acute-toned, grave, dull, or sonorous; sometimes they are of short duration, resembling the chirping of small birds, or the noise of a dry sucker passing through the pipe of a pump. These varieties may co-exist, or follow in quick succession. They arise from a collection of mucous matter, not considerable in quantity, but very viscous. When the stethoscope is applied directly over the place where the rattles occur, a slight vibration is communicated to the instrument. This is not perceived if the point where the rattles exist is far from the cylinder. The sound leads one to imagine that the rattles arise from the bubbling of a liquid, like what takes place when we blow with a pipe into a solution of soap, such as boys use to make air-balloons; and we fancy, too, that the ear can detect a difference in the bulk of those bubbles, (which variation probably arises from a greater or less viscosity of the liquid;) and hence the rattles are termed *large*, *very large*, *middling*, *small*, and *minute*. Mucous rattles are generally large; crepitation, small.

4. *Exploration of the movements of the heart.*—The alternate contractions of the ventricles and auricles of the heart produce sounds very distinct, and of a different character. They should be examined in four principal relations: 1°, the extent to which they may be heard by the aid of the cylinder; 2°, the shock, or the impulsive force, of the organ; 3°, the nature and intensity of the sound; 4°, the rhythm according to which the different parts of the heart contract. The extent of the pulsations of the heart should be considered in two respects: that of the first sensation which the use of the cylinder gives rise to

* This is one of the species of organic lesion, of which no account is given in any author previous to M. Laennec. The reader must wait for the description of it, until we meet with it in our regular course through the pathological part of the work.

when applied to the precordial region, and that of points of the chest, besides this region, where the beating of the heart may be discerned. In the healthy state, that organ produces such a sensation, that it appears evidently to correspond to a small extent of the thoracic parietes, and hardly to pass beyond the point to which the instrument is applied. Sometimes it appears to be totally covered by the cylinder, and profoundly situate in the mediastinal cavity, so as to leave a vacant space between itself and the sternum; and its movements, though energetic, do not produce any apparent vibration in the adjacent parts. In other cases, on the contrary, it seems entirely to fill this cavity; and its contractions, though slow and without noise, appear to raise the anterior paries of the chest to a great extent, and to cause a great divergence of the viscera internally: this sensation seems to indicate a more or less unusually voluminous heart. In the healthy state, the pulsations do not extend beyond the precordial region; the motions of the left cavities are perceived under the cartilages of the fifth and seventh ribs of the left side; those of the right cavities, under the sternum: when this bone is short, the pulsations extend into the epigastrium. In very fat persons, and in those in whom the beating of the heart cannot be felt by the hand, the space in which they can be heard by the aid of the cylinder is sometimes confined to a surface of about an inch square. In meagre persons, in those who have a narrow chest, and even in children, the pulsations occupy a greater space, extending sometimes throughout the whole length of the sternum, to the anterior and superior part of the left side of the chest as far as the clavicle, and sometimes, but less sensibly, under the right clavicle. When the pulsations are confined to this space, and they are less sensible under the clavicles than in the precordial region, in persons of the habit of body above indicated, the heart is well formed. When the extent of the pulsations becomes more considerable, these may be heard: 1°, on the left side of the chest, from the axilla to the corresponding region of the stomach; 2°, on the right side, to the same extent; 3°, at the posterior part of the left side of the chest; 4°, lastly, but rarely, at the right posterior part. The intensity of the sound is less according to the order of the parts indicated. The pulsations of the aorta and subclavian arteries are not sensible, except in cases of aneurism. The extent of the beating of the heart is in a direct ratio to the weakness and thinness of its parietes, and *vice versâ*. The intensity of the shock, or sense of percussion, communicated to the ear by the cylinder, is, in general, in an inverse ratio of the extent of the pulsations of the heart, and in a direct ratio of the thickness of the parietes of the ventricles. In a man whose heart is constructed in the manner most favourable to the free exercise of the circulation, this impulsion is but little remarkable, and often even insensible, especially if the person be somewhat fat. The alternative contractions of the different parts of the heart are distinctly heard by the aid of the cylinder. In the natural state, this sound is double, and each beat of the arteries corresponds to two successive sounds: one clear and sudden, analogous to the rap of the valve of a bellows, corresponding to the systole of the auricles; the other more dull, more prolonged, isochronous with the pulsation of the arteries, as well as with the sensation of the shock formerly described, and which indicates the contraction of the ventricles. In the natural state, the sounds of the contractions of the two sides of the heart are similar. When this organ is too full of blood, the cylinder transmits only a dull sort of roaring sound, very similar to the noise made by a pair of bellows, and, if it becomes stronger, to that made by filing wood. The rhythm, or order, of the pulsations in the healthy state, is as follows: At the moment when the artery strikes the finger, the ear receives an impulse, with a rather dull, though distinct, sound: this is due to the contraction of the ventricles. Immediately afterwards, a sharper sound, analogous to that of the crack of a whip, the valve of a bellows, or the lapping of a dog, announces the contraction of the auricles. No motion sensible to the ear accompanies this noise, no interval of repose separates it from that of the contraction of the ventricles. The duration of the sound from the auricles is less than that from the ventricles. After the systole of the latter, the heart falls for an instant into a state of absolute immobility. The respective duration of the contractions of the auricles and ventricles may, then, be determined in the following manner: Of the duration of the contractions of the heart, one-third at most, or even only one-fourth, is occupied by the systole of the auricles; one-fourth, or a little less, by absolute repose; and the half, or nearly so, by the systole of the ventricles.*

### Medical and Philosophical Intelligence.

* Trentell and Würtz, booksellers, of Soho-square, import the stethoscope from Paris, (Paris price, two francs:) the instrument is also made by Allnut, a turner, of Piccadilly, London.

